# CYLD Maintains Retinal Homeostasis by Deubiquitinating ENKD1 and Promoting the Phagocytosis of Photoreceptor Outer Segments

**DOI:** 10.1002/advs.202404067

**Published:** 2024-10-07

**Authors:** Song Yang, Fan Yu, Mulin Yang, Hua Ni, Weiwen Bu, Hanxiao Yin, Jia Yang, Weishu Wang, Denghui Zhai, Xuemei Wu, Nan Ma, Te Li, Huijie Hao, Jie Ran, Ting Song, Dengwen Li, Sei Yoshida, Quanlong Lu, Yunfan Yang, Jun Zhou, Min Liu

**Affiliations:** ^1^ Department of Genetics and Cell Biology College of Life Sciences State Key Laboratory of Medicinal Chemical Biology Haihe Laboratory of Cell Ecosystem Nankai University Tianjin 300071 China; ^2^ School of Health and Life Sciences Qingdao Central Hospital University of Health and Rehabilitation Sciences Qingdao 266113 China; ^3^ Center for Cell Structure and Function Shandong Provincial Key Laboratory of Animal Resistance Biology College of Life Sciences Shandong Normal University Jinan 250014 China; ^4^ Department of Cell Biology School of Basic Medical Sciences Cheeloo College of Medicine Shandong University Jinan 250012 China; ^5^ Laboratory of Tissue Homeostasis Haihe Laboratory of Cell Ecosystem Tianjin 300462 China

**Keywords:** CYLD, deubiquitination, ENKD1, phagocytosis, photoreceptor, retinal pigment epithelium

## Abstract

Phagocytosis of shed photoreceptor outer segments by the retinal pigment epithelium (RPE) is essential for retinal homeostasis. Dysregulation of the phagocytotic process is associated with irreversible retinal degenerative diseases. However, the molecular mechanisms underlying the phagocytic activity of RPE cells remain elusive. In an effort to uncover proteins orchestrating retinal function, the cylindromatosis (CYLD) deubiquitinase is identified as a critical regulator of photoreceptor outer segment phagocytosis. CYLD‐deficient mice exhibit abnormal retinal structure and function. Mechanistically, CYLD interacts with enkurin domain containing protein 1 (ENKD1) and deubiquitinates ENKD1 at lysine residues K141 and K242. Deubiquitinated ENKD1 interacts with Ezrin, a membrane‐cytoskeleton linker, and stimulates the microvillar localization of Ezrin, which is essential for the phagocytic activity of RPE cells. These findings thus reveal a crucial role for the CYLD‐ENKD1‐Ezrin axis in regulating retinal homeostasis and may have important implications for the prevention and treatment of retinal degenerative diseases.

## Graphical abstract

In this study, Yang et al. demonstrate that CYLD deubiquitinates ENKD1 in RPE cells and thereby promotes ENKD1 interaction with Ezrin. This interaction facilitates Ezrin localization to the microvilli of RPE cells, stimulating their phagocytosis of shed photoreceptor outer segments. These findings reveal a critical role for the CYLD‐ENKD1‐Ezrin axis in maintaining retinal homeostasis.

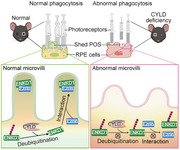

## 1 Introduction

Retinal degenerative diseases causing visual loss have become a major public health burden.^[^ ^1^ ^]^ The retinal pigment epithelium (RPE) is a monolayer of regular polygonal cells occupying the outermost layer of the retina. On its outer surface, the RPE is associated with the Bruch's membrane and the choroid, while on its inner surface, it is connected to the outer segment (OS) of photoreceptor cells.^[^ ^2^ ^]^ Continuous photoreceptor cell renewal results in the shedding of part of the apical outer segment, which must be phagocytosed and cleared by RPE cells to maintain the normal visual cycle.^[^ ^3^ ^]^ Abnormal phagocytosis leads to anomalous renewal of photoreceptor OS (POS), a key driver of age‐related macular degeneration (AMD), one of the most common causes of blindness worldwide.^[^ ^4^ ^]^ Currently, there are no effective treatments for retinal degenerative diseases related to the dysfunction of RPE.^[^ ^5^ ^]^ Hence, there is an urgent need to investigate the mechanisms regulating the phagocytic function of RPE cells to provide insights for the development of preventive and therapeutic interventions.

Each RPE cell connects with ≈30 photoreceptor cells and is responsible for the clearance of their shed POS, which makes RPE cells one of the most proficient phagocytic cell types in nature.^[^ ^6^ ^]^ Phagocytosis is facilitated by the raised microvilli at the RPE cell surface, which create a microvillous space in which the tips of POS are embedded.^[^ ^2^, ^7^ ^]^ Ezrin, a cytoskeleton‐related protein, localizes to the apical microvilli of RPE cells and links actin filaments to the plasma membrane, thereby playing a crucial role in the formation and maintenance of microvilli. Decrease in Ezrin expression or its mislocalization significantly reduces the phagocytic capacity of RPE cells.^[^ ^8^ ^]^ Therefore, a normal microvillus structure is critical for the phagocytic function of RPE cells.

Increasing evidence has shown that dysregulation of protein ubiquitination is a major cause of hereditary and idiopathic neurodegenerative diseases, including AMD.^[^ ^4^, ^9^ ^]^ In addition, the ubiquitination system has been implicated in the phagocytotic activity of RPE cells.^[^ ^10^ ^]^ In humans, there are dozens of deubiquitinases responsible for removing ubiquitin modifications from proteins.^[^ ^11^ ^]^ The cylindromatosis (CYLD) deubiquitinase mainly cleaves lysine 63 (K63)‐linked polyubiquitin chains from its substrates, thereby regulating protein‐protein interactions and subcellular localization.^[^ ^12^ ^]^ The deubiquitinating activity of CYLD is abolished by replacing the conserved catalytic residue, cysteine 601, with serine, which is referred to as CYLD C/S.^[^ ^5^, ^13^ ^]^

Previous studies have demonstrated that CYLD regulates various signaling pathways, including nuclear factor‐кB, Wnt/β‐catenin, and transforming growth factor‐β signaling, by modulating the assembly of protein complexes.^[^ ^14^ ^]^ Our previous findings showed that CYLD interacts with the microtubule cytoskeleton through direct and indirect mechanisms, thereby modulating multiple cellular processes, including cell polarization, cell migration, cell junction assembly, and ciliogenesis.^[^ ^13^, ^15^ ^]^ One of our recent studies revealed that CYLD participates in the regulation of hearing, a physiological process closely associated with aging.^[^ ^16^ ^]^ However, it remains unknown whether CYLD plays a role in other age‐related neurological disorders, such as retinal degenerative diseases.

In this study, we identified *CYLD* as the most significantly down‐regulated deubiquitinase gene in the retina of aged individuals. CYLD deficiency in aged mice led to impaired RPE phagocytic function and retinal degeneration. We then demonstrated that CYLD promotes the phagocytic function of RPE through deubiquitinating enkurin domain containing protein 1 (ENKD1), a centrosome/microtubule‐associated protein that regulates spindle orientation and ciliogenesis.^[^ ^17^ ^]^ Further mechanistic studies revealed that CYLD‐mediated deubiquitination of ENKD1 facilitates its interaction with Ezrin, which is essential for the maintenance of the microvillar structure and phagocytic function of RPE.

## 2 Results

### 2.1 CYLD Depletion Triggers Retinal Degeneration

**Figure 1:**
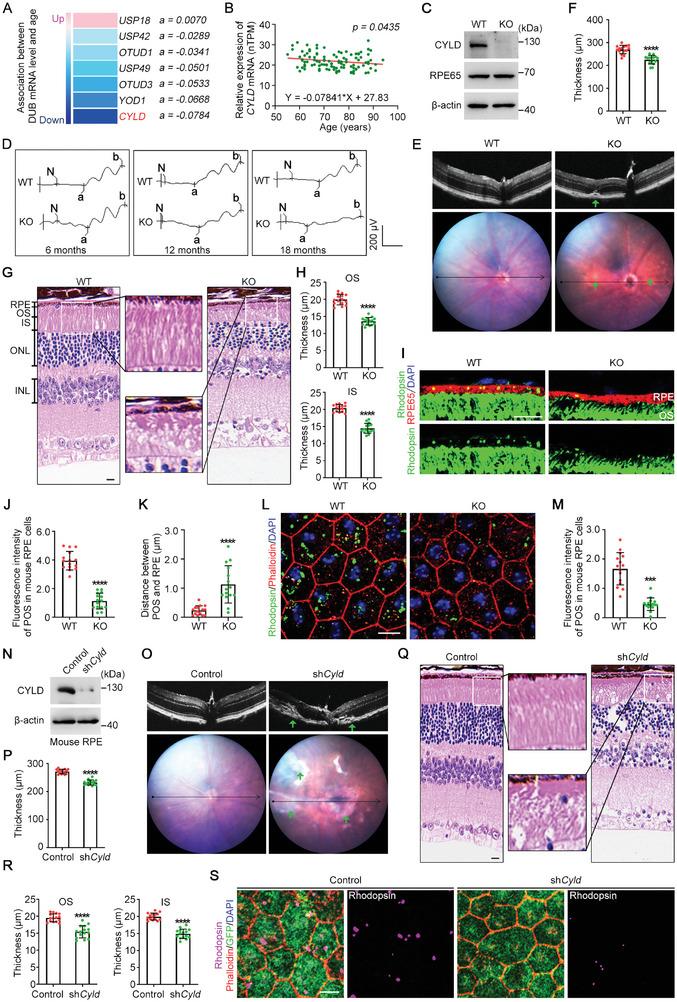
Loss of CYLD causes retinal degeneration and impairs POS phagocytosis. A) Association between deubiquitinatase (DUB) mRNA level and age. DUB transcriptional levels in human retina were obtained from the Human Protein Atlas. The “a” is slope value of the standard curve Y (DUB mRNA level) = aX (age) +b, indicating the degree of change. B) Association between *Cyld* mRNA level and age. C) Immunoblotting of WT and *Cyld* KO mouse eyes. D) WT and *Cyld* KO mice (6‐, 12‐, and 18‐month‐old) were examined by electroretinography. E,F) WT and *Cyld* KO mice were examined with a Micron IV retinal imaging microscope (E), and the retinal thickness was measured (F, n = 15 fields from 5 mice). The green arrow indicates a possible site of retinopathy. G) Retinal structures of WT and *Cyld* KO mice examined by H&E staining. Scale bar, 20 µm. H) The thicknesses of OS (top) and IS (bottom) layers (n = 15 fields from 5 mice). I‐K) Immunofluorescence microscopy of frozen sections of WT and *Cyld* KO mouse retinas to determine the phagocytic function of RPE cells (M). Scale bar, 10 µm. The fluorescence intensity of the engulfed POS in RPE cell (N) and the distance from the RPE layer to POS (O) were quantified (n = 15 fields from 5 mice). L, M) Horizontal views of the RPE layer of WT and *Cyld* KO mouse retinas to show the phagocytic function of RPE cells (P). The fluorescence intensity of the engulfed POS was quantified (Q, n = 15 fields from 5 mice). Scale bar, 10 µm. N Immunoblotting of the RPE in mice injected with control or sh*Cyld* AAVs. O, P) Mice injected with control or sh*Cyld* AAVs were examined with a retinal imaging microscope (O), and the retinal thickness was measured (P, n = 15 fields from 5 mice). Q,R) Retinal structures of mice injected with control or sh*Cyld* AAVs were examined by H&E staining (Q, Scale bar, 20 µm). The thickness of OS (top) and IS (bottom) layers is shown in R (n = 15 fields from 5 mice). S) Horizontal views of the RPE layer to show the phagocytic function of RPE cells in mice injected with control or sh*Cyld* AAVs. Scale bar, 10 µm. ns, not significant, *** *p* < 0.001, **** *p* < 0.0001.

To systemically evaluate the involvement of the ubiquitination system in retinal function, the transcriptional levels of 96 deubiquitinases in the retinas of over 100 human individuals were obtained from the Human Protein Atlas (www.proteinatlas.org). Analysis of these data showed that the transcriptional levels of 7 deubiquitinases in the retina were significantly related to age (**Figure** **1**A). Among them, the level of the deubiquitinase CYLD in the retina tends to decline with age (Figure 1B).

We thus hypothesized that CYLD may be involved in age‐related retinal degeneration. To test this hypothesis, whole‐body *Cyld* knockout (KO) mice were used, and the efficiency of CYLD depletion was determined by immunoblotting of mouse retina (Figure 1C; Figure S1A, Supporting Information). Electroretinography (ERG) was then performed on wild‐type (WT) and *Cyld* KO mice at 6, 12, and 18 months of age. ERG measurements revealed that at 18 months of age, the a‐wave of the response, which reflects the general physiological activity of photoreceptors in the outer retina, and the b‐wave, which reflects the inner layer activity of ON bipolar cells and Müller cells, were both significantly decreased in *Cyld* KO mice compared to WT mice (Figure 1D; Figure S1B,C, Supporting Information). Microscopic analysis of the ocular fundus revealed clear signs of retinal degenerative lesions and significantly reduced retinal thickness in 18‐month‐old *Cyld* KO mice (Figure 1E,F). These findings suggest that loss of CYLD may contribute to the development of retinal degeneration.

To further determine the role of CYLD in the retina, the retinal structures of 18‐month‐old WT and *Cyld* KO mice were examined by hematoxylin and eosin (H&E) staining. The results showed that the OS and inner segment (IS) of photoreceptor cells in *Cyld* KO mice were structurally disorganized and significantly thinner than those of WT mice (Figure 1G,H; Figure S1D, Supporting Information). Additionally, the outer nuclear layer (ONL) and inner nuclear layer (INL) were also significantly thinner in *Cyld* KO mice (Figure S1E, Supporting Information). The nuclear density was significantly decreased in the ONL and INL of *Cyld* KO mice (Figure S1F, Supporting Information). Meanwhile, the retina of 6‐ and 12‐month‐old *Cyld* KO mice showed no obvious structural defects (Figure S1G,H, Supporting Information). These results thus indicate an age‐related retinopathy in *Cyld* KO mice.

RPE cells contribute to the maintenance of photoreceptor renewal by phagocytosing shed POS, thereby protecting normal retinal structure and function. To determine the potential involvement of aberrant POS phagocytosis in the retinal degeneration observed in *Cyld* KO mice, the retinas of 18‐month‐old WT and *Cyld* KO mice were analyzed 1 hour after light onset. Firstly, vertical sections of the RPE layer were imaged, and the results showed a significant reduction in engulfed POS in *Cyld* KO mice compared with WT mice (Figure 1I,J; Figure S1I, Supporting Information). In addition, an increased distance between RPE and POS was also observed in *Cyld* KO mice (Figure 1K), indicating an impaired association between the RPE layer and photoreceptors. Horizontal views of the RPE layer also showed a significant reduction in engulfed POS in *Cyld* KO mice (Figure 1L,M).

To further explore the link between CYLD deficiency‐induced retinal degeneration and the phagocytic function of RPE cells, adeno‐associated viruses (AAVs) carrying *Cyld* shRNAs (sh*Cyld*), along with Rpe65 and GFP elements, were generated to achieve RPE‐specific knockdown of CYLD in mice. In 12‐month‐old mice, a month after subretinal injection of the sh*Cyld* AAVs, the level of CYLD in the RPE was significantly reduced (Figure 1N). Microscopic analysis of the ocular fundus revealed that CYLD knockdown in the RPE resulted in retinal degeneration, marked by notable thinning of the retina (Figure 1O,P). H&E staining of the retinal tissue further indicated that the thickness of the OS, IS, ONL, and INL was reduced, with decreased nuclear density in the ONL and INL. This phenotype mirrored that observed in *Cyld* KO mice (Figure 1Q,R; Figure S1J–L, Supporting Information). Additionally, phagocytic function assays of RPE cells showed a marked reduction in the phagocytic activity following CYLD knockdown (Figure 1S; Figure S1M, Supporting Information). In summary, the above results suggest that CYLD may play a crucial role in retinal homeostasis by enhancing the phagocytic activity of RPE cells.

### 2.2 CYLD Promotes RPE‐Mediated POS Phagocytosis

**Figure 2:**
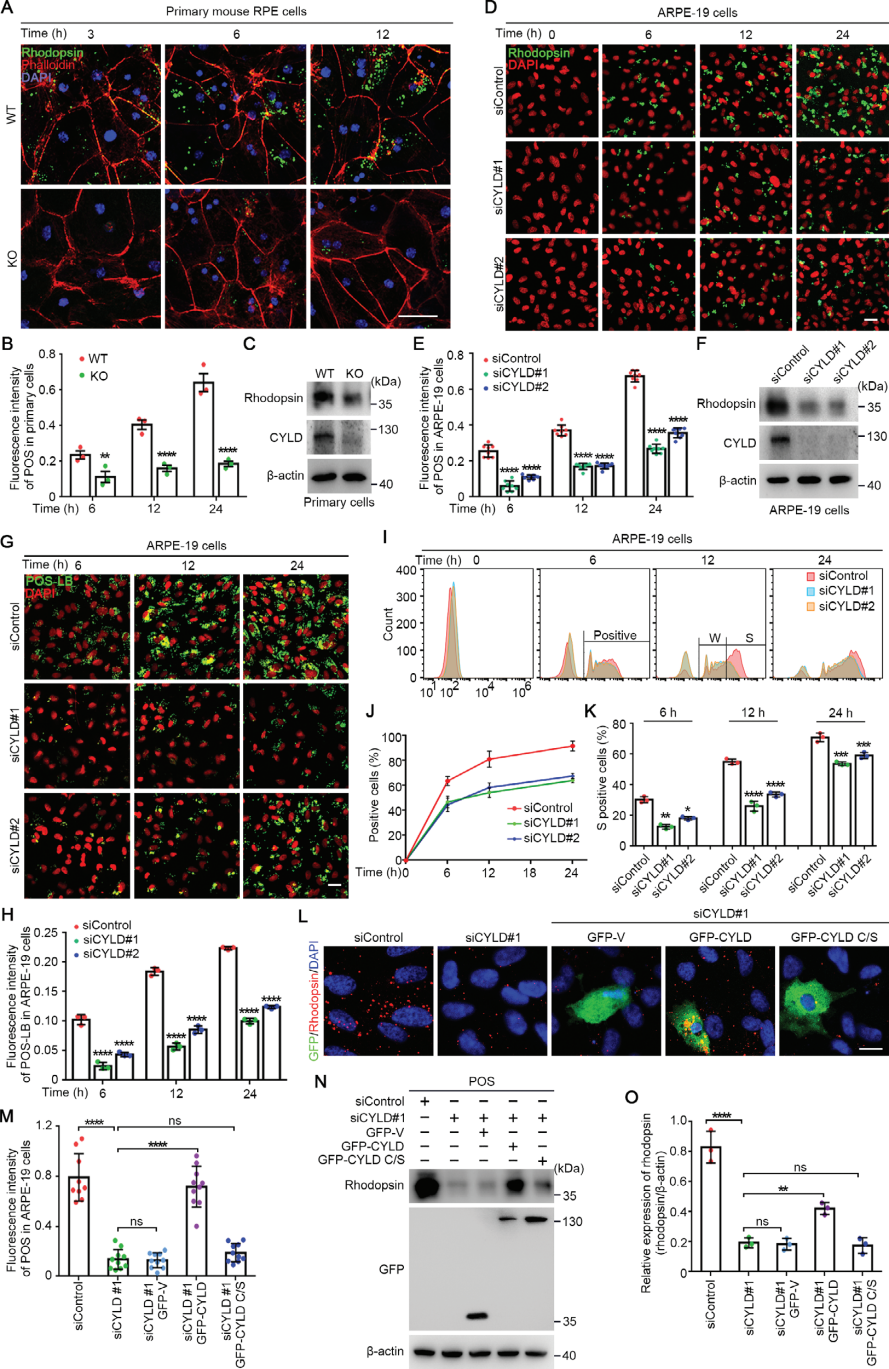
CYLD promotes the phagocytic function of RPE cells through its deubiquitinase activity. A, B) Immunofluorescence microscopy of POS phagocytosis by primary RPE cells isolated from WT and *Cyld* KO mice (A). The fluorescence intensity of engulfed POS in RPE cells was quantified (B, n = 3 fields, from three independent experiments). Scale bar, 10 µm. C) Immunoblotting of POS phagocytosis by primary RPE cells isolated from WT and *Cyld* KO mice. D, E) Immunofluorescence images of control or CYLD siRNA‐treated ARPE‐19 cells phagocytizing POS (D). The fluorescence intensity of engulfed POS was quantified (E, n = 8 fields, from three independent experiments). Scale bar, 10 µm. F) Immunoblotting‐based analysis of POS phagocytosis by control or CYLD siRNA‐treated ARPE‐19 cells. G, H) Immunofluorescence‐based analysis of POS phagocytosis by control or CYLD siRNA‐treated ARPE‐19 cells. ARPE‐19 cells were transfected with siRNAs for 48 h and incubated with POS‐LB for indicated periods. Cells were subjected to immunofluorescence microscopy (G), and the fluorescence intensity of engulfed POS‐LB was quantified (H, n = 9 fields, from three independent experiments). Scale bar, 10 µm. I‐K) Flow cytometry‐based analysis of POS phagocytosis in control or CYLD siRNA‐treated ARPE‐19 cells. The fluorescence intensity of engulfed POS‐LB in each cell was detected by flow cytometry (I). Weak fluorescence (W) represents cells with weak phagocytic activity. Strong fluorescence (S) represents cells with strong phagocytic activity. POS‐LB‐positive cells (J) and cells with strong fluorescence (K) were quantified. L–O) Immunofluorescence‐based analysis of POS phagocytosis by ARPE‐19 cells treated with control or CYLD siRNAs for 24 h and then overexpressed with GFP, GFP‐CYLD, or GFP‐CYLD C/S. Cells were incubated with POS for 12 h and then subjected to immunofluorescence microscopy (L) and immunoblotting (N). The fluorescence intensity of engulfed POS was quantified (M, n = 9 cells). The intensity of rhodopsin signal was quantified (O). Scale bar, 10 µm. ns, not significant, **p < 0.01, ****p < 0.0001.

To determine the role of CYLD in RPE‐mediated POS phagocytosis, primary RPE cells were isolated from WT and *Cyld* KO mouse retinas (Figure S2A,B, Supporting Information) and then cultured with POS extract. Immunofluorescence microscopy of POS engulfment at different time points showed a significant reduction in the fluorescence quantity of POS engulfed by *Cyld* KO RPE cells compared with WT RPE cells (**Figure** **2**A,B). This observation was further confirmed by immunoblotting of the POS marker rhodopsin in RPE cells (Figure 2C). POS phagocytosis was also performed using human adult RPE‐19 (ARPE‐19) cells, in which CYLD knockdown significantly reduced the ability of ARPE‐19 cells to engulf POS (Figure 2D–F). To further validate the above findings, POS‐coated fluorescent latex beads (POS‐LB) were used to evaluate the phagocytic ability of in vitro cultured cells. Immunofluorescence microscopy revealed that primary *Cyld* KO RPE cells had a reduced capacity to phagocytose POS‐LB (Figure S2C,D, Supporting Information). Similarly, CYLD depletion significantly suppressed the ability of ARPE‐19 cells to engulf POS‐LB (Figure 2G,H). To further quantify the phagocytic activity, we performed flow cytometry. ARPE‐19 cells were classified into weak and strong groups based on the varying levels of POS‐LB uptake, as indicated by the fluorescence intensity. Quantitative analysis was then conducted separately for the total positive cells and the strongly positive cells. By this flow cytometry‐based method, we also found significantly fewer fluorescence‐positive ARPE‐19 cells (containing engulfed POS‐LB) in CYLD siRNA‐treated groups compared to the control siRNA‐treated group (Figure 2I–K). These findings thus demonstrate that CYLD promotes the phagocytic activity of RPE cells.

Next, we sought to determine whether CYLD‐mediated regulation of POS phagocytosis depends on its deubiquitinase activity. ARPE‐19 cells were treated with CYLD siRNA, overexpressed with GFP‐tagged CYLD or an enzymatically deficient CYLD mutant (cysteine 601 mutated to serine, C/S), and cultured with POS. Immunofluorescence microscopy revealed that overexpression of CYLD, but not its enzymatically deficient mutant, significantly rescued the phagocytic activity of CYLD siRNA‐treated cells (Figure 2L,M). These findings were further validated by immunoblotting of rhodopsin in the above cells (Figure 2N,O). A phagocytosis assay using POS‐LB also showed that the deubiquitinase activity of CYLD is essential for POS phagocytosis in ARPE‐19 cells (Figure S2E,F, Supporting Information). Together, these results demonstrate that CYLD promotes RPE‐mediated POS phagocytosis in a deubiquitinase‐dependent manner.

### 2.3 CYLD Interacts with ENKD1

**Figure 3:**
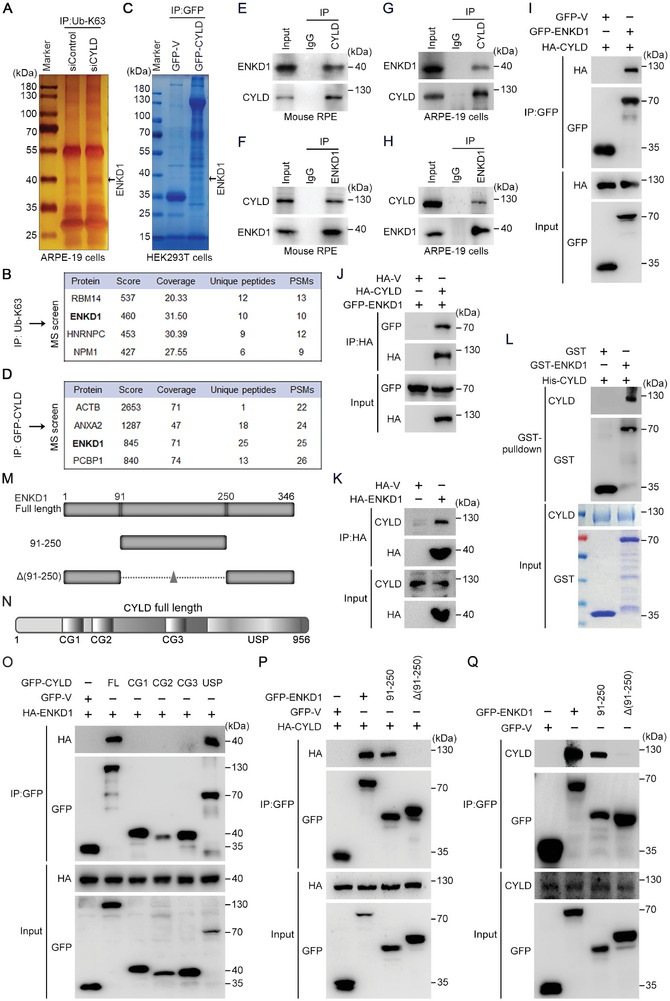
CYLD interacts with ENKD1. A,B) Immunoprecipitation (IP) of K63‐linked polyubiquitinated proteins in ARPE‐19 cells treated with control or CYLD siRNAs. Silver staining (A) and mass spectrometry (B) were used for protein analysis. C, D) IP analysis of K63‐linked polyubiquitinated proteins in HEK293T cells overexpressed with GFP or GFP‐CYLD for 24 h. Coomassie blue staining (C) and mass spectrometry (D) were used for protein analysis. E, F) IP analysis to detect the interaction between endogenous CYLD and ENKD1 in mouse RPE tissues. G, H) IP analysis to detect the interaction between endogenous CYLD and ENKD1 in ARPE‐19 cells. I, J) IP analysis to detect the interaction between exogenous CYLD and ENKD1 in HEK293T cells. K) IP analysis to detect the interaction between endogenous CYLD and exogenous ENKD1 in HEK293T cells. L) GST‐pulldown to detect the interaction between purified CYLD and ENKD1. M, N) Fragments and domains of human ENKD1 (M) and CYLD (N) used for this study. O) IP analysis mapping domains of CYLD for ENKD1 binding. P, Q) IP analysis mapping domains of ENKD1 for exogenous (P) and endogenous (Q) CYLD binding.

We next explored the molecular mechanism by which CYLD regulates POS phagocytosis. Specifically, we sought to identify the substrate(s) of CYLD involved in this process. Immunoprecipitation and subsequent mass spectrometry of K63 linkage‐specific polyubiquitinated proteins in ARPE‐19 cells suggested that ENKD1 is a potential substrate of CYLD (**Figure** **3**A,B). In addition, mass spectrometry of CYLD‐binding proteins in human embryonic kidney 293T (HEK293T) cells also revealed a possible interaction between CYLD and ENKD1.

To determine whether CYLD regulates RPE phagocytosis through ENKD1, we knocked down ENKD1 in ARPE‐19 cells and evaluated their ability to engulf POS by immunofluorescence microscopy and immunoblotting. Indeed, ENKD1 knockdown significantly reduced the ability of ARPE‐19 cells to engulf POS (Figure S3A–E, Supporting Information). Consistently, flow cytometry also showed significantly fewer fluorescence‐positive ARPE‐19 cells (containing engulfed POS‐LB) in ENKD1 siRNA‐treated groups compared to the control siRNA‐treated group (Figure S3F–H, Supporting Information). Thus, ENKD1, a potential novel substrate of CYLD, is also required for POS phagocytosis.

To further establish the interaction between CYLD and ENKD1, co‐immunoprecipitation of endogenous CYLD and ENKD1 was performed, and the results showed that CYLD interacts with ENKD1 in ARPE‐19 cells (Figure 3E,F). Next, exogenous CYLD and ENKD1 with the indicated tags were overexpressed in HEK293T cells. Co‐immunoprecipitation assays showed a robust interaction between CYLD and ENKD1 (Figure 3G,H). In HEK293T cells, endogenous CYLD also interacts with exogenously expressed HA‐tagged ENKD1 (Figure 3I). Furthermore, an in vitro GST‐pulldown assay using purified His‐tagged CYLD and GST‐tagged ENKD1 showed a direct interaction between CYLD and ENKD1 (Figure 3J). To define which domains in ENKD1 and CYLD are required for their interaction, full‐length and truncated ENKD1 and CYLD (Figure 3K,L) were expressed in HEK293T cells. Co‐immunoprecipitation assays demonstrated that the CYLD‐ENKD1 interaction is dependent on the ubiquitin‐specific protease (USP) domain of CYLD and the 91–250 amino acid residues of ENKD1 (Figure 3M–O).

### 2.4 CYLD Deubiquitinates ENKD1 at K141 and K242

**Figure 4:**
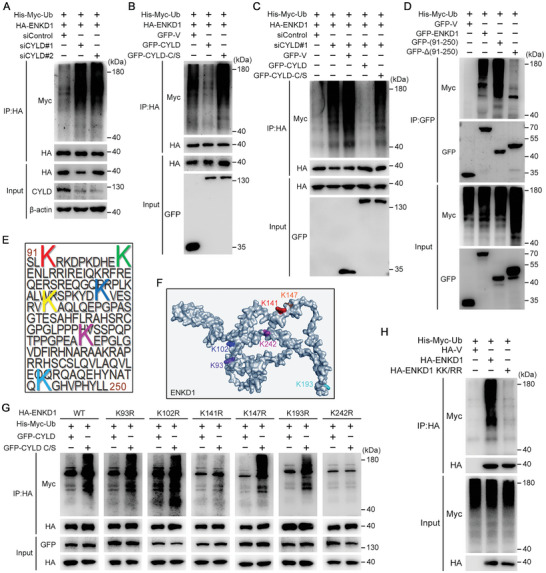
CYLD deubiquitinates ENKD1 at K141 and K242. A) IP analysis of HA‐ENKD1 polyubiquitination in HEK293T cells transfected with control or CYLD siRNAs. B) IP analysis of HA‐ENKD1 polyubiquitination in HEK293T cells overexpressed with GFP, GFP‐CYLD, or GFP‐CYLD C/S. C) IP analysis of HA‐ENKD1 polyubiquitination in HEK293T cells transfected with control or CYLD siRNAs and then overexpressed with GFP, GFP‐CYLD, or GFP‐CYLD C/S. D) IP analysis of the polyubiquitination level of ENKD1 truncations. E) Potential ubiquitination sites on exogenously expressed GFP‐ENKD1 (91‐250 aa) identified by mass spectrometry. F) Localization of potential ubiquitination sites on ENKD1. The structure of ENKD1 was generated by AlphaFold2. G) IP analysis of the polyubiquitination levels of ENKD1 and ENKD1 mutants in HEK293T cells overexpressed with GFP‐CYLD or GFP‐CYLD C/S. H) IP analysis of the polyubiquitination levels of HA‐ENKD1 and the HA‐ENKD1 K141R/K242R (KK/RR) mutant in HEK293T cells.

To establish ENKD1 as a substrate of CYLD, the expression of CYLD in HEK293T cells was manipulated, and the change in ENKD1 ubiquitination was assessed by immunoprecipitation. Overexpression of CYLD, but not its enzymatically deficient mutant, significantly reduced the level of ENKD1 ubiquitination, while CYLD knockdown significantly enhanced ENKD1 ubiquitination (**Figure** **4**A,B). In addition, exogenously expressed CYLD, but not its enzymatically deficient mutant, significantly ablated the enhancement of ENKD1 ubiquitination by CYLD depletion (Figure 4C).

To map out the ubiquitination site(s) on ENKD1, we examined the ubiquitination level of full‐length and truncated forms of ENKD1. The results revealed that the majority of the polyubiquitination sites on ENKD1 reside in the domain containing 91–250 amino acid residues (Figure 4D). Mass spectrometry also identified 6 potential ubiquitination sites in this domain (Figure 4E,F). Single‐site mutants of ENKD1 were then constructed and expressed together with CYLD or its enzymatically deficient mutant. Immunoprecipitation revealed that CYLD catalyzes ENKD1 deubiquitination mainly at two lysine residues, K141 and K242 (Figure 4G). A double‐site mutation on ENKD1 (K141R/K242R, KK/RR) largely ablated its overall polyubiquitination, demonstrating that K141 and K242 are the two major sites for ENKD1 polyubiquitination (Figure 4H).

### 2.5 CYLD is Required for the Formation of RPE Microvilli

**Figure 5:**
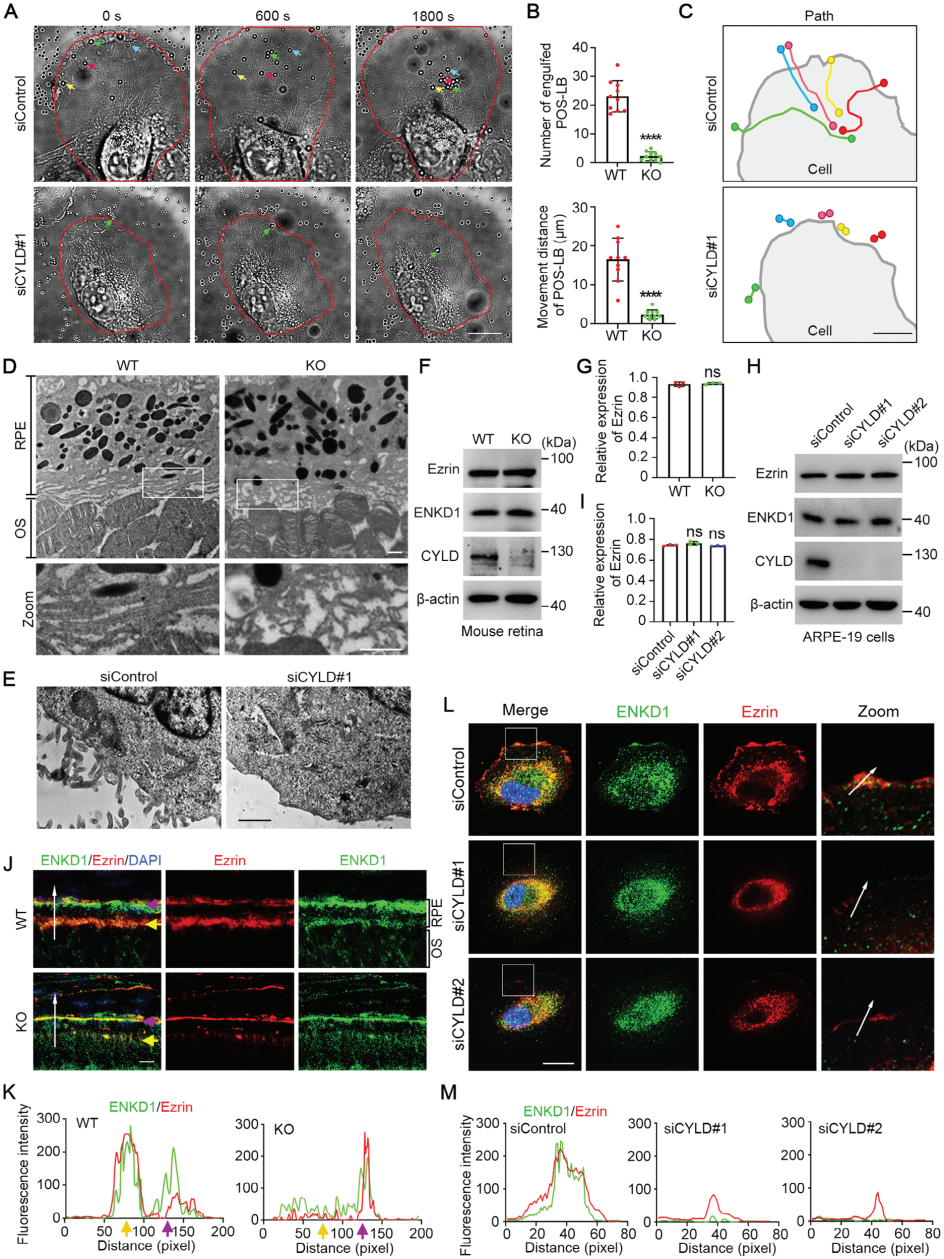
CYLD promotes the formation of RPE microvilli. A‐C) Live‐cell images showing the engulfment of POS‐LB by ARPE‐19 cells treated with control or CYLD siRNAs. Cell boundaries are highlighted with red lines. Selected POS‐LB were labeled with colored arrows (A). Engulfed POS‐LB number and movement distance of POS‐LB were quantified (B, n = 9 cells). The movement paths of selected POS‐LB were plotted using ImageJ (C). D) Transmission electron microscopy showing the microvillar structure in 18‐month‐old WT and *Cyld* KO mouse retinas. Scale bar, 1 µm. E) Transmission electron microscopy showing the microvillar structure on ARPE‐19 cells treated with control or CYLD siRNA. Scale bar, 1 µm. F,G) Immunoblotting (F) and quantification (G) of Ezrin expression in WT and *Cyld* KO mouse retinas. H,I) Immunoblotting (H) and quantification (I) of Ezrin expression in cultured ARPE‐19 cells treated with control or CYLD siRNA. J, K) Immunofluorescence images of WT and *Cyld* KO mouse retinas showing the localization of ENKD1 (green) and Ezrin (red) (J). The colocalization of ENKD1 and Ezrin (along the white arrow) was analyzed (K). Yellow arrows indicate the apical surface of RPE. Purple arrows indicate the basal surface of RPE. Scale bar, 10 µm. L,M) Immunofluorescence images of RPE‐19 cells transfected with control or CYLD siRNAs (L). The colocalization of ENKD1 (green) and Ezrin (red) at the cell cortex (along the white arrow) was analyzed (M). Scale bar, 10 µm. ns, not significant, ****p < 0.0001.

To understand the mechanism underlying the role of the CYLD‐ENKD1 axis in POS phagocytosis, live‐cell imaging was used to examine the entry of POS‐LB into ARPE‐19 cells. The results showed that the fluorescence quantity of engulfed POS‐LB and the average movement distance of POS‐LB were both significantly reduced in CYLD‐depleted cells (**Figure** **5**A,B). Consistently, the movement path analysis also revealed that CYLD is essential for the entry of POS‐LB into ARPE‐19 cells (Figure 5C). These findings indicate that CYLD may participate in the initiation of POS phagocytosis.

RPE cells form microvillar structures, which play an essential role in facilitating the entry of shed POS into RPE cells. Therefore, the effect of CYLD depletion on RPE microvilli was determined. Transmission electron microscopy showed that, compared to WT RPE cells, the microvilli of *Cyld* KO RPE cells were markedly disorganized (Figure 5D). Similarly, the microvilli were also defective in CYLD‐depleted ARPE‐19 cells (Figure 5E). Previous studies indicated that the RPE microvillar assembly and function are dependent on the proper expression and subcellular localization of Ezrin.^[^ ^8a,b^ ^]^ Our analysis of mouse retinal tissues and cultured ARPE‐19 cells showed that CYLD depletion had no significant effect on the expression of Ezrin (Figure 5F–I). We then examined the subcellular localization of Ezrin and found that in *Cyld* KO mice, ENKD1 and Ezrin failed to localize to RPE microvilli (Figure 5J,K). Consistently, in cultured ARPE‐19 cells, CYLD knockdown largely abolished the cortical localization of ENKD1 and Ezrin (Figure 5L,M). Therefore, CYLD deficiency impairs RPE microvilli, possibly due to a disruption in the subcellular localization of ENKD1 and Ezrin.

### 2.6 CYLD Promotes the Interaction between ENKD1 and Ezrin

**Figure 6:**
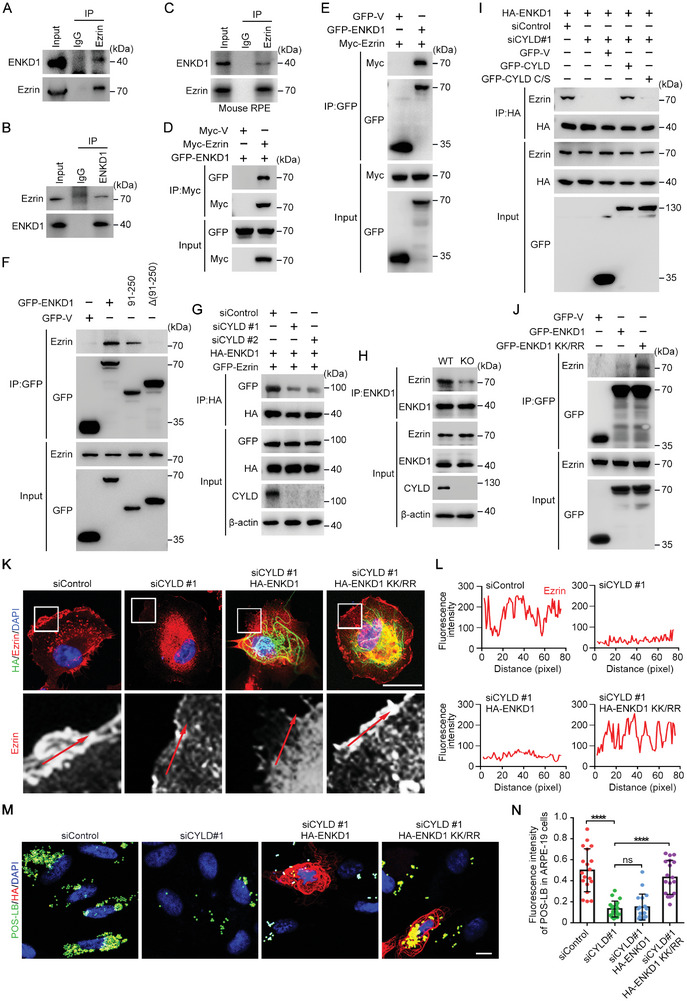
CYLD promotes the ENKD1‐Ezrin interaction by deubiquitinating ENKD1. A, B) IP analysis to detect the interaction between endogenous ENKD1 and Ezrin in ARPE‐19 cells. C) IP analysis to detect the interaction between endogenous ENKD1 and Ezrin in mouse RPE tissues. D, E) IP analysis to detect the interaction between exogenous ENKD1 and Ezrin in HEK293T cells. F) IP analysis to map domains of ENKD1 for Ezrin binding. G) IP analysis of ENKD1‐Ezrin binding affinity in HEK293T cells transfected with control or CYLD siRNAs. H) IP analysis of ENKD1‐Ezrin binding affinity in RPE cells from WT and *Cyld* KO mouse retinas. I) IP analysis of ENKD1‐Ezrin interaction in HEK293T cells transfected with control or CYLD siRNAs and then overexpressed with GFP, GFP‐CYLD, or GFP‐CYLD C/S. J) IP analysis (I) and quantification (J) of Ezrin binding to ENKD1 and ENKD1 KK/RR. K, L) Immunofluorescence images of ARPE‐19 cells transfected with control or CYLD siRNAs and then overexpressed with HA‐ENKD1 or HA‐ENKD1 KK/RR (K). The fluorescence intensity of Ezrin at the cell cortex (along the red arrow) was quantified (L). Scale bar, 10 µm. M, N) Immunofluorescence‐based analysis of POS phagocytosis by ARPE‐19 cells transfected with control or CYLD siRNA and then overexpressed with HA‐ENKD1 or HA‐ENKD1 KK/RR (M). The fluorescence intensity of engulfed POS‐LB was quantified (N, n = 15 cells). Cells were incubated with POS‐LB for 24 h before analysis. Scale bar, 10 µm. ns, not significant; ****p < 0.0001.

We then sought to examine whether ENKD1 interacts with Ezrin. Co‐immunoprecipitation of endogenous ENKD1 and Ezrin was performed, and the results showed that ENKD1 interacts with Ezrin in cultured ARPE‐19 cells (**Figure** **6**A,B). We performed using mouse retinal tissue lysates also revealed an interaction between ENKD1 and Ezrin (Figure 6C). Next, exogenous ENKD1 and Ezrin with the indicated tags were overexpressed in HEK293T cells. Co‐immunoprecipitation showed a robust interaction between CYLD and ENKD1 (Figure 6D,E). To define which domain(s) of ENKD1 is required for its interaction with Ezrin, full‐length and truncated forms of ENKD1 were expressed in HEK293T cells. Co‐immunoprecipitation assays demonstrated that the ENKD1‐Ezrin interaction is dependent on the 91–250 amino acid residues of ENKD1 (Figure 6F).

Since we have demonstrated that CYLD interacts with ENKD1 and deubiquitinates ENKD1 at K141 and K242, we then evaluated whether the interaction between ENKD1 and Ezrin is regulated by CYLD. Co‐immunoprecipitation demonstrated that CYLD depletion attenuated the interaction between ENKD1 and Ezrin (Figure 6G). Similar results were observed in mouse tissues, where the interaction between ENKD1 and Ezrin was weakened in the RPE of *Cyld* KO mice (Figure 6H). Further analysis showed that overexpression of CYLD, but not its enzymatically deficient mutant, rescued the impaired ENKD1‐Ezrin interaction in CYLD siRNA‐treated HEK293T cells (Figure 6I). In addition, compared to ENKD1, the ubiquitination‐deficient mutant ENKD1 KK/RR had a significantly increased binding affinity for Ezrin (Figure 6J). These data thus demonstrate that CYLD promotes the interaction between ENKD1 and Ezrin by deubiquitinating ENKD1 at K141 and K242.

Next, the functional relevance of CYLD‐mediated ENKD1 deubiquitination in POS phagocytosis was determined. Immunofluorescence microscopy showed that the ubiquitination‐deficient mutant ENKD1 KK/RR, but not ENKD1, significantly rescued the impaired cortical localization of Ezrin in CYLD siRNA‐treated ARPE‐19 cells (Figure 6K,L). Moreover, overexpression of ENKD1 KK/RR, but not ENKD1, significantly restored the phagocytic capability of CYLD‐depleted RPE cells (Figure 6M,N). Therefore, our results demonstrate that CYLD deubiquitinates ENKD1 and promotes its interaction with Ezrin, leading to enhanced Ezrin microvillar localization and POS phagocytosis.

## 3 Discussion

**Figure 7:**
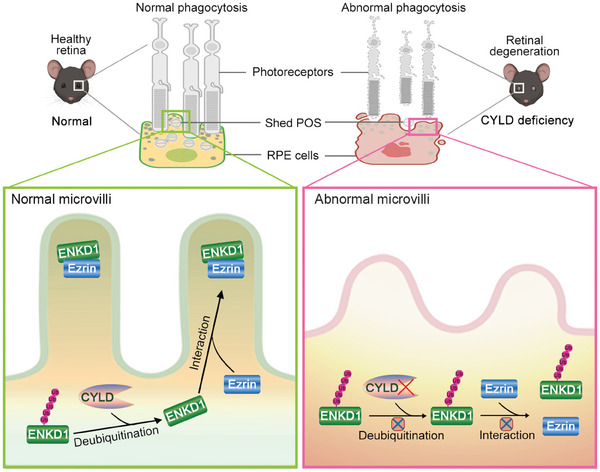
Molecular model for the function of CYLD in regulating the phagocytic activity of RPE cells. CYLD interacts with and deubiquitinates ENKD1, strengthening its interaction with Ezrin and promoting Ezrin localization to the microvilli of RPE cells. Loss of CYLD disrupts the above pathway and impairs the microvillar structure and the phagocytic activity of RPE cells, ultimately driving retinal degeneration.

In this study, we have identified CYLD as a critical regulator of the phagocytic function of RPE cells. Further investigation into the molecular mechanism reveals that CYLD interacts with and deubiquitinates ENKD1, thereby enhancing its interaction with Ezrin and facilitating the proper localization of Ezrin to microvilli. This process is essential for maintaining the normal structure of microvilli and the phagocytic function of RPE cells (**Figure** **7**). When CYLD is absent, the level of ENKD1 ubiquitination increases, weakening its interaction with Ezrin and preventing Ezrin localization to microvilli. As a result, the structural integrity and functionality of microvilli are compromised, leading to impaired phagocytic activity of RPE cells (Figure 7). Overall, the disruption of this regulatory mechanism due to CYLD deficiency damages the microvilli of RPE cells, suppresses the phagocytosis of POS, and ultimately contributes to retinal degeneration. Our findings thus provide new insights into the molecular mechanism of POS phagocytosis and offer new opportunities for the prevention and treatment of retinal degenerative diseases.

Phagocytosis, first described by Elie Metchnikoff over a century ago, is a key component of both innate and adaptive immune responses to pathogen invasion.^[^ ^18^ ^]^ Professional phagocytes such as neutrophils, macrophages, and dendritic cells, engulf pathogens such as bacteria and fungi to eliminate them from the site of infection.^[^ ^19^ ^]^ It is worth noting that phagocytosis was initially associated with the homeostatic processes of nutrient uptake and tissue resorption. RPE cells, for example, are specialized phagocytes that play a critical role in the renewal of photoreceptor cells and maintenance of visual function, and they engulf more material than any other cell type during their lifecycle.^[^ ^4^, ^20^ ^]^ In addition to phagocytosis, RPE cells perform a number of other essential functions, such as maintaining the blood‐tissue barrier, accumulating vitamin A, and transporting glucose and cholesterol.^[^ ^2^, ^21^ ^]^

CYLD has three N‐terminal cytoskeleton‐associated protein glycine‐rich (CAP‐Gly) domains and a C‐terminal USP domain. Through the USP domain, CYLD can specifically remove K63‐linked polyubiquitin chains from its substrates, thereby regulating a variety of cellular activities by modulating protein subcellular localization and protein‐protein interaction. Through its first two CAP‐Gly domains, CYLD interacts with microtubules and promotes the dynamics and stability of microtubules.^[^ ^12^, ^15^ ^]^ Previously, we demonstrated that CYLD participates in RhoA‐mediated cytoskeletal rearrangement by deubiquitinating leukemia‐associated RhoGEF.^[^ ^22^ ^]^ In this study, we found that the deubiquitinase activity of CYLD contributes to RPE microvillar assembly and POS phagocytosis. Whether CYLD participates in the regulation of other actin‐based structures or processes may warrant further investigation.

Extensive research has been conducted on the function of CYLD in immune system development and immune regulation.^[^ ^23^ ^]^ In professional phagocytic macrophages, for instance, CYLD has been shown to modulate the inflammatory response and cell death. A recent study also revealed that CYLD is involved in transient receptor potential A1 (TRPA1)‐mediated melanosome phagocytosis in keratinocytes.^[^ ^24^ ^]^ Further research is required to determine if and how the CYLD‐ENKD1‐Ezrin axis is involved in these processes.

Phagocytosis of POS by RPE cells can be divided into three stages: binding, transport, and digestion. The shed POS is captured by receptors on the microvilli of RPE cells before being invaginated by the microvillar membrane to form phagocytic cups. Subsequently, the POS enters RPE cells, forming phagosomes that are transported to lysosomes to form phagolysosomes in a microtubule‐dependent manner.^[^ ^25^ ^]^ Ultimately, these components are digested and metabolized.^[^ ^3^, ^26^ ^]^ RPE cells are unable to engulf POS in the absence of CYLD, which is associated with the disruption of microvilli on the RPE cell surface. Previous studies have demonstrated that the externalization of phosphatidylserine on the membranes of shed POS serves as an “eat me” signal, which must be recognized by receptors on the surface of RPE cells to initiate the phagocytic cascade. Several signaling pathways are involved in this recognition process, including the well‐established Mer receptor tyrosine kinase (MerTK) and αVβ5 integrin pathways.^[^ ^2^, ^27^ ^]^ Dysregulation of these pathways can impair the phagocytic function of RPE cells, potentially leading to retinal degeneration. Notably, the signal‐receiving proteins are primarily localized on the microvillar membranes of RPE cells. If these microvilli are damaged, shortened, or reduced in number, the receptors on their surface are distanced from the “eat me” signal, thereby hindering the efficiency of the phagocytic process. The microvillar structure is present on the apical surface of various types of epithelial cells, including those lining the small intestine.^[^ ^28^ ^]^ Thus, it would not be surprising if the disruption of the CYLD‐ENKD1‐Ezrin axis is found to result in structural abnormalities of microvilli in other tissues as well.

RPE cell structure and function are essential for normal vision, whereas alterations in RPE can impair retinal function and lead to retinopathy.^[^ ^2^, ^29^ ^]^ Preserving the microvillar structure of RPE cells facilitates phagocytosis and reduces the risk of retinal diseases.^[^ ^2^, ^29^ ^]^ Despite our increased understanding of the cell biology and molecular genetics of retinal degenerative diseases, there are currently no preventative or therapeutic treatments. The present study has identified CYLD‐mediated ENKD1 deubiquitination as a molecular mechanism that controls POS phagocytosis. Thus, targeting this molecular pathway may provide new opportunities for the prevention and treatment of related retinal diseases.

## 4 Experimental Section

### Animals

The reproduction and genotyping of *Cyld* knockout (KO) mice are described previously.^[^ ^30^ ^]^ Briefly, *Cyld* heterozygous mice were intercrossed to generate WT and *Cyld* KO littermates. The construction strategy of mice *Cyld* KO mice were shown in Figure S1A, Supporting Information. To achieve CYLD knockdown specifically in the RPE of mice, AAVs carrying *Cyld* shRNAs, along with Rpe65 and GFP elements, were generated by Shanghai Genechem. AAVs containing Rpe65 and GFP elements were used as control. The AAVs were injected into the subretinal space of 12‐month‐old mice, which were then maintained for a month before retinal analysis was performed. For POS phagocytosis analysis, mice were euthanized 1 h after light onset. All animal studies were approved by the Animal Care and Use Committee of Nankai University (2024‐SYDWLL‐000645), and mice were housed and operated in accordance with relevant regulations.

### Cell Culture

HEK293T cells were obtained from the American Type Culture Collection (ATCC, Manassas, VA) and were cultured in Dulbecco's modified Eagle medium (DMEM) supplemented with 10% fetal bovine serum (FBS, C04001‐500, VivaCell Biosciences, Shanghai, China). ARPE‐19 cells were purchased from the China Center for Type Culture Collection (CCTCC, Shanghai, China) and were cultured in DMEM/F12 1:1 medium with 10% FBS. For phagocytosis analysis, ARPE‐19 cells were cultured for up to 3 months in DMEM/F12 with pyruvate (11360‐070, Gibco) as described before.^[^ ^31^ ^]^ Primary RPE cells were isolated and cultured as described.^[^ ^32^ ^]^ All cells were cultured in a humidified incubator containing 5% CO_2_ at 37 °C.

### ERG Tests

The mice were kept in darkness for 12 h beforehand, and all tests were performed under dim red light to maintain dark adaptation. Mice were anesthetized by intraperitoneal injection with 2.5% avertin. The pupils were dilated using one drop of 0.5% tropicamide phenylephrine, and one drop of 0.5% oxybuprocaine hydrochloride was administered for local anesthesia. The reference electrodes of the visual electrophysiological system (RetiMINER‐C, IRC Technologies) were then placed subcutaneously below the ears. The ground electrode was placed on the tail, and the recording electrodes were placed on the corneal surface of each eye. Scotopic ERG responses to white flash stimuli (3 cd s m^−2^) were recorded. The a‐wave amplitude was measured from the baseline to the a‐wave trough, and the b‐wave amplitude was measured from the a‐wave trough to the b‐wave peak.

### H&E Staining

The eyeballs of mice were removed and fixed in 4% paraformaldehyde overnight at 4 °C. The cornea and lens were then removed, and the retinas were fixed for an additional 2 h. The retinas were embedded with paraffin, sectioned at a thickness of 4 µm, and subjected to H&E staining (G1120, Solarbio). To ensure that the sections used for imaging came from the same eccentricity, the eyecups were embedded in the same orientation. To eliminate errors caused by measuring different retinal positions and to ensure accurate measurement of retinal thickness, sections were made along the axis of the optic nerve and the center of the cornea. After H&E staining, retinal thickness was measured at equal distance from the optic nerve to ensure the scientific reliability of the data.

### Antibodies and Chemicals

The following primary antibodies were purchased from indicated sources: anti‐CYLD (SAB4200060, Sigma‐Aldrich); anti‐ENKD1 (ab224561, Abcam); anti‐Ezrin (610 603, BD Biosciences); anti‐RPE65 (ab231782, Abcam); anti‐rhodopsin (ab5417, Abcam); anti‐β‐actin (ab8226, Abcam); anti‐GFP (11 814 460 001, Roche); anti‐HA (H3663, Sigma‐Aldrich); anti‐Myc (m4439, Sigma‐Aldrich); anti‐GST (G7781, Sigma‐Aldrich). Horseradish peroxidase‐conjugated secondary antibodies were purchased from Solarbio Science & Technology. Alexa Fluor 488 and 568 secondary antibodies were from Life Technologies. Anti‐GFP magnetic beads (ab193983, Abcam) and anti‐HA magnetic beads (B26202, Bimake) were purchased from Abcam and Bimake, respectively. DAPI (D5942, Sigma‐Aldrich) and TRITC‐phalloidin (P1951, Sigma‐Aldrich) were purchased from Sigma‐Aldrich.

### Immunoblotting and Immunoprecipitation

For immunoblotting, samples were lysed using the RIPA cell lysis buffer (50 mM Tris‐HCl, 1% Triton, 0.1% SDS, 1% sodium deoxycholate, 150 mM NaCl, and 1 mM EDTA, pH 7.5), boiled in the SDS loading buffer, and resolved by SDS‐PAGE. For immunoprecipitation, samples were lysed using the immunoprecipitation buffer (20 mM Tris‐HCl, 150 mM NaCl, 1 mM EDTA, 1 mM EGTA, 1% NP‐40, 2.5 mM sodium pyrophosphate, 10% glycerol, pH 7.5) supplemented with protease inhibitor cocktail (4 693 159 001, Roche). Lysates were then incubated with antibody‐conjugated agarose beads at 4 °C for 4 h, washed five times with the immunoprecipitation buffer, and analyzed by immunoblotting.

### RNAi and Plasmids

CYLD and ENKD1 siRNAs were purchased from Guangzhou RiboBio (Guangzhou, China). The sequences for CYLD siRNAs were 5′‐CGAAGAGGCUGAAUCAUAA‐3′ (#1) and 5′‐GAUUGUUACUUCUAUCAAA‐3′ (#2), and sequences for ENKD1 siRNAs were 5′‐GAAGGACCCTAAGGACCAT‐3′ (#1) and 5′‐GGCCCAAAGTCTTCGTGAA‐3′ (#2). All siRNAs were transfected into cells using Lipofectamine RNAiMAX (13 778 030, Invitrogen). Mammalian expression plasmids for GFP‐CYLD, GFP‐CYLD C/S, HA‐CYLD, GFP‐ENKD1, HA‐ENKD1, GFP‐Ezrin, Myc‐Ezrin, and ENKD1 truncations and mutants were generated by inserting PCR fragments into plasmid vectors or through site‐directed mutagenesis. All constructs were confirmed by DNA sequencing. Plasmids were transfected into HEK293T cells using polyethylenimine (PEI, 23966‐1, Polysciences Inc.) and into ARPE‐19 cells using Lipofectamine 3000 (L3000‐015, Invitrogen).

### Phagocytosis Assay

POS extraction and separation were performed according to a modified Parinot protocol.^[^ ^33^ ^]^ In brief, pig eyes obtained from the slaughterhouse were placed on ice and in darkness. The eye was cut in half, and the lens and part of the neuroretinal layer were removed. Then, the remaining pink portion was carefully removed from the optic cup and placed into a mass balancing solution. After further coarse filtration with two layers of gauze, samples were placed under continuous conditions of 4 °C and a sucrose gradient of 20–60% before centrifugation in a Beckman SW‐27/SW‐32‐TI oscillating rotor at 25 000 rpm for 1 h at 4 °C. The appropriate retinal layer was collected, centrifuged at 5000 rpm for 10 min, and cleaned three times. The final POS extractions were resuspended in DMEM and stored at −80 °C. POS‐coated fluorescent latex beads (POS‐LB) were prepared according to the previous method.^[^ ^34^ ^]^ In brief, 15 mg POS protein and 10 µl fluorescent latex beads (L4655, Sigma‐Aldrich) were mixed and incubated for 4 h at 4 °C to obtain POS‐LB. To analyze phagocytotic function, the cells were cultured to become monolayer, and then POS or POS‐LB were added to the cell culture medium, and then RPE cells were analyzed at the indicated time points.

### Fluorescence Microscopy

Cultured cells and tissue sections were fixed with 4% paraformaldehyde (PFA, P6148, Sigma‐Aldrich) for 30 min and permeabilized with 0.5% Triton X‐100 (Merck KGaA) for 15 min. Then samples were blocked with 4% bovine serum albumin (BSA, A1128) in PBS for 1 h before being incubated with primary antibodies, secondary antibodies, and DAPI. A Zeiss LSM710 confocal microscope was then used to examine the stained cells and tissue sections. The imaging was performed using a z‐stack mode to capture fluorescence signals throughout the entire cell or tissue as comprehensively as possible. To evaluate the phagocytic function of mouse RPE cells or ARPE‐19 cells, confocal images with fluorescence overlays were analyzed. Phagocytic activity was quantified by calculating the average fluorescence intensity per cell, defined as: Phagocytic capacity = Fluorescence intensity per unit area / Number of cells per unit area. The fluorescence intensity was quantified using ImageJ software (National Institutes of Health). For live‐cell imaging, ARPE‐19 cells were seeded in live‐cell culture dishes, treated as indicated, and incubated with POS‐LB for 3 h. Time‐lapse images of cells and POS‐LB were captured with a 633 objective lens (DMI 6000, Leica Microsystems). The TrackMate plugin of Fiji software (National Institutes of Health) was used for analysis.

### Flow Cytometry

Cultured cells were treated with POS‐LB as described above and incubated at 37 °C. After incubation for the indicated time, the trypsin digestion was terminated by adding DMEM medium containing 10% serum. The cells were then harvested and washed three times with PBS. Cells not incubated with POS‐LB were used as a negative control. The cells were fixed in 4% paraformaldehyde for 20 min, followed by washing with PBS and filtration to prepare the samples for analysis. The samples were immediately analyzed using an LSRFortessa Multicolor Flow Cytometer (BD), with 5000 events collected per sample. Data were analyzed using the FlowJo software (Tree Star).

### Transmission Electron Microscopy

Fresh mouse optic cup tissue and cell pellets were fixed in 2.5% glutaraldehyde (G5882, Sigma‐Aldrich), post‐fixed in 2% osmium tetroxide, dehydrated in graded ethanol and propylene oxide, embedded in Epon resin for 24 h at 60 °C. Ultrathin sections (50 nm) were then prepared, mounted on 200 mesh copper grids, double‐stained with uranyl acetate and lead citrate, and examined using a transmission electron microscope (Hitachi HT7700 Exalens).

### Statistical Analysis

The data were presented as mean ± SD and analyzed using GraphPad Prism software (GraphPad Software, La Jolla, CA). Unpaired two‐tailed Student's *t*‐test was used to compare two groups. ANOVA with post hoc tests was used to compare multiple groups. For statistical analysis of three data points, the Mann‐Whitney non‐parametric test was utilized. P‐values under 0.05 were considered to be statistically significant.

## Conflict of Interest

The authors declare no conflict of interest.

## Author Contributions

Y.Y., J.Z., and M.L. supervised the project. S.Y., J.Z., and M.L. designed experiments. S.Y., F.Y., M.Y., H.N., W.B., H.Y., J.Y., W.W., D.Z., X.W., N.M., T.L., H.H., J.R., and T.S. performed experiments. S.Y., F.Y., D.L., S.Y., Q.L., Y.Y., J.Z., and M.L. analyzed data. S.Y., Q.L., Y.Y., J.Z., and M.L. wrote the manuscript.

## Supporting information



Supporting Information

## Data Availability

The data that support the findings of this study are available from the corresponding author upon reasonable request.
