# Supplementary material for: CYLD Maintains Retinal Homeostasis by Deubiquitinating ENKD1 and Promoting the Phagocytosis of Photoreceptor Outer Segments
Source: Adv Sci (Weinh). 2024 Oct 7;11(45):2404067. doi: 10.1002/advs.202404067 (PMC11615780; doi:10.1002/advs.202404067)
Supplement: Supplementary file 1 — Supporting Information [file ADVS-11-2404067-s001.docx]

**Supporting Information**

**CYLD maintains retinal homeostasis by deubiquitinating ENKD1 and promoting the phagocytosis of photoreceptor outer segments**

Song Yang^1,2^, Fan Yu^1,2^*, Mulin Yang^1^, Hua Ni^1^, Weiwen Bu^1^, Hanxiao Yin^1^, Jia Yang^1^, Weishu Wang^1^, Denghui Zhai^1^, Xuemei Wu^1^, Nan Ma^1^, Te Li^1^, Huijie Hao^1^, Jie Ran^3^, Ting Song^3^, Dengwen Li^1^, Sei Yoshida^1^, Quanlong Lu^1^*, Yunfan Yang^4^*, Jun Zhou^1,3^*, Min Liu^5^*

^1^Department of Genetics and Cell Biology, College of Life Sciences, State Key Laboratory of Medicinal Chemical Biology, Haihe Laboratory of Cell Ecosystem, Nankai University, Tianjin 300071, China.

^2^School of Health and Life Sciences, Qingdao Central Hospital, University of Health and Rehabilitation Sciences, Qingdao 266113, China.

^3^Center for Cell Structure and Function, Shandong Provincial Key Laboratory of Animal Resistance Biology, College of Life Sciences, Shandong Normal University, Jinan 250014, China.

^4^Department of Cell Biology, School of Basic Medical Sciences, Cheeloo College of Medicine, Shandong University, Jinan 250012, China.

^5^Laboratory of Tissue Homeostasis, Haihe Laboratory of Cell Ecosystem, Tianjin 300462, China.

*Correspondence: yufan@uor.edu.cn (F.Y.); 9920220142@nankai.edu.cn (Q.L.); yunfanyang@sdu.edu.cn (Y.Y.); junzhou@nankai.edu.cn (J.Z.); minliu@nankai.edu.cn (M.L.)

Running title: CYLD promotes the phagocytosis of photoreceptor outer segments

**
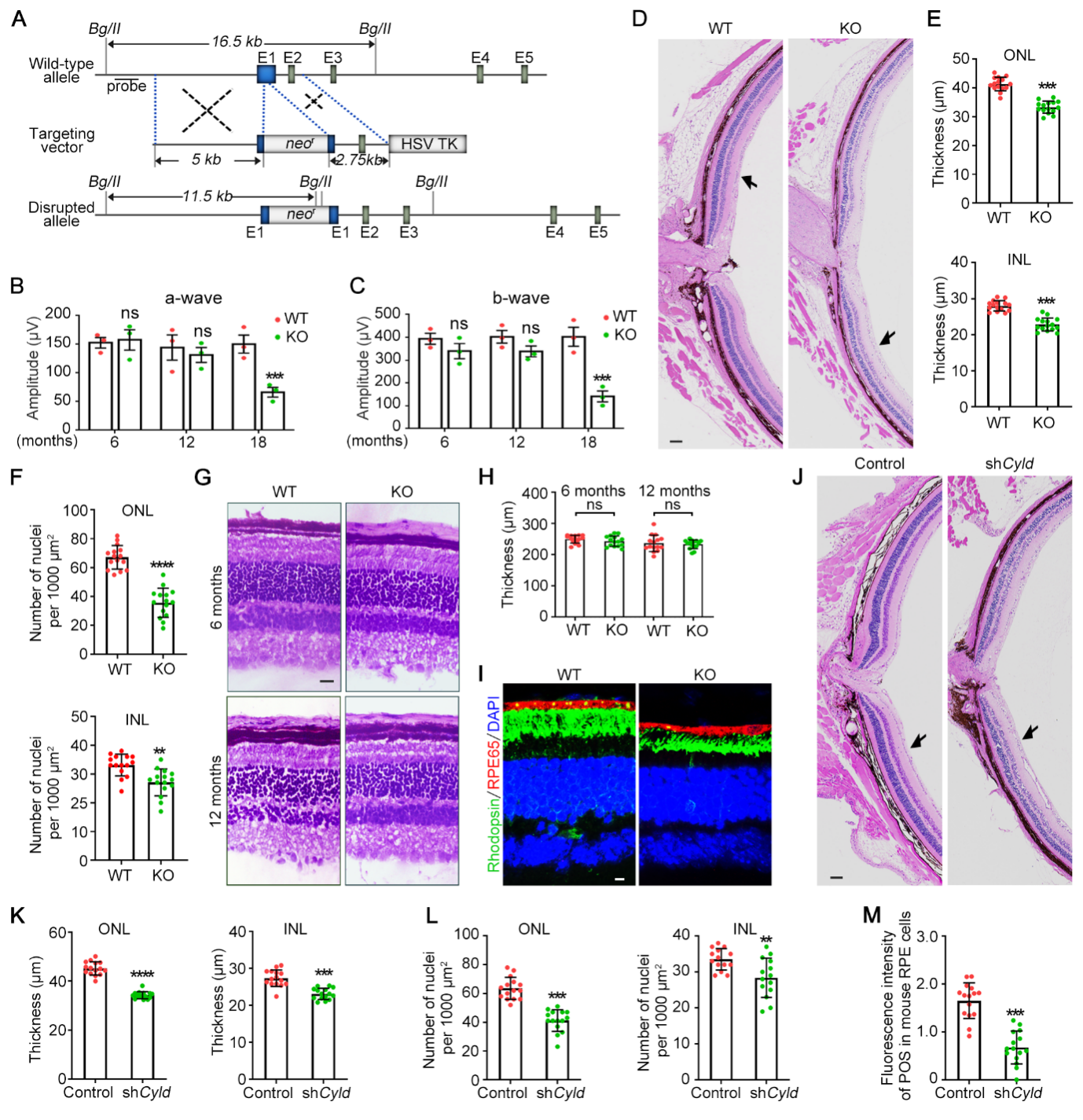
**

**Figure S1 Construction and examination of *Cyld* KO mice.**

**A** Strategy for the generation of *Cyld* KO mice. **B, C** WT and *Cyld* KO mice (6-, 12-, and 18-month-old) were examined by electroretinography, and the amplitudes of a-wave (B) and b-wave (C) were quantified, n = 3 mice. **D** Retinal structures of WT and *Cyld* KO mice examined by H&E staining. The black arrow indicates the enlarged area in Figure 1G. Scale bar, 50 μm. **E** The thicknesses of ONL (top) and INL (bottom) layers (n = 15 fields from 5 mice) of WT and *Cyld* KO mouse retinas were measured. **F** Analysis of nuclear densities in ONL (top) and INL (bottom) of WT and *Cyld* KO mouse retinas, n = 15 fields from 5 mice. **G, H** H&E staining of retina sections of 6- and 12-month-old WT and *Cyld* KO mice (G). Scale bar, 20 μm. Retinal thickness was measured (H, n = 15 fields from 5 mice). **I** Retinal structures of WT and *Cyld* KO mice examined by immunofluorescence microscopy. Scale bar, 10 μm. **J** Retinal structures of Control and sh*Cyld* mice examined by H&E staining. The black arrow indicates the enlarged area in Figure 1Q. Scale bar, 50 μm. **K** The thicknesses of ONL (left) and INL (right) layers (n = 15 fields from 5 mice) of Control and sh*Cyld* mouse retinas were measured. **L** Analysis of nuclear densities in ONL (left) and INL (right) of Control and sh*Cyld* mouse retinas, n = 15 fields from 5 mice. **M** The fluorescence intensity of the engulfed POS was quantified of Figure 1S. ns, not significant, ** *p* < 0.01, *** *p* < 0.001, **** *p* < 0.0001.


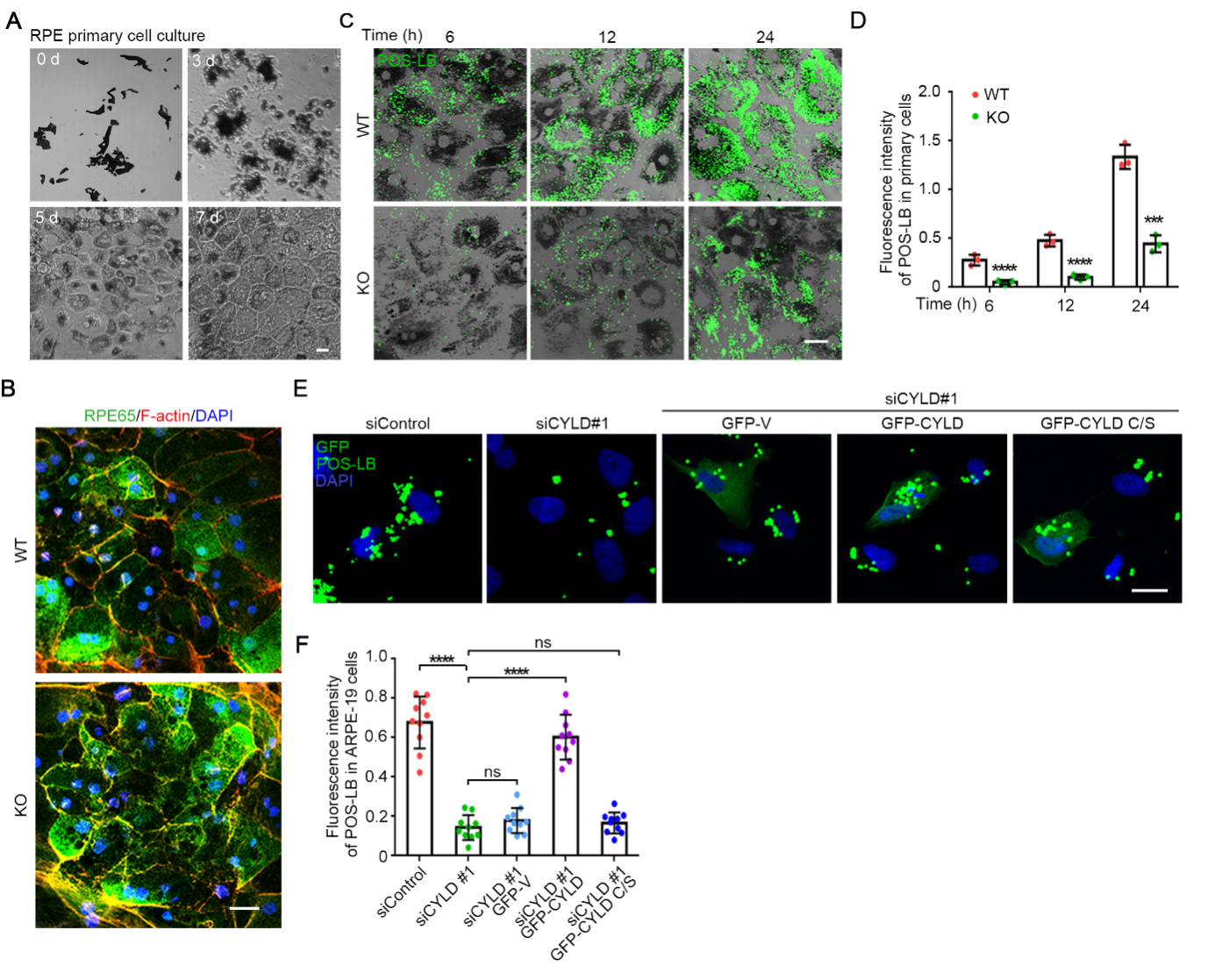


**Figure S2 CYLD depletion impairs POS phagocytosis.**

**A** Optical microscopy of primary mouse RPE cells. **B** Immunofluorescence images of primary RPE cells from WT and *Cyld* KO mice. **C, D** Immunofluorescence-based analysis of POS phagocytosis by primary RPE cells from WT and *Cyld* KO mice (C). The fluorescence intensity of engulfed POS-LB was quantified (D). **E, F** Immunofluorescence-based analysis of POS phagocytosis by ARPE-19 cells treated with control or CYLD siRNAs and then overexpressed with GFP, GFP-CYLD, or GFP-CYLD C/S (E). The fluorescence intensity of engulfed POS-LB was quantified (F). All experiments were repeated at least three times. Scale bar, 10 μm. ns, not significant; *** *p* < 0.001, **** *p* < 0.0001.

**
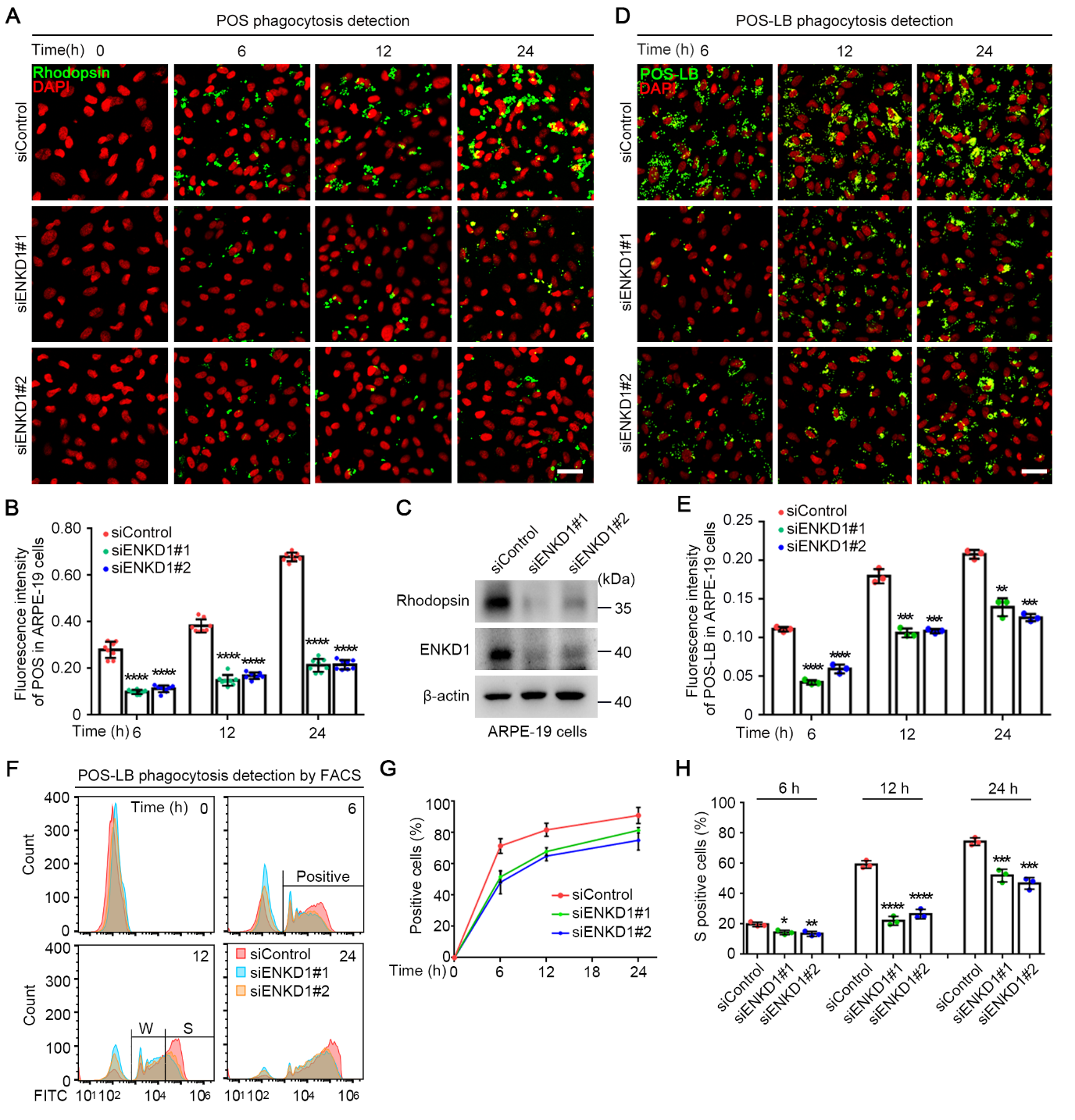
**

**Figure S3 ENKD1 depletion impairs POS phagocytosis.**

**A, B** Immunofluorescence-based analysis of POS phagocytosis by ARPE-19 cells treated with control or ENKD1 siRNAs (A). The fluorescence intensity of engulfed POS was quantified (B). **C** Immunoblotting-based analysis of POS phagocytosis by ARPE-19 cells treated with control or ENKD1 siRNAs. **D, E** Immunofluorescence-based analysis of POS phagocytosis by control or ENKD1 siRNA-treated ARPE-19 cells (D). The fluorescence intensity of engulfed POS-LB was quantified (E). **F-H** Flow cytometry-based analysis of POS phagocytosis by ARPE-19 cells treated with control or ENKD1 siRNAs. The fluorescence intensity of engulfed POS-LB in each cell was detected by flow cytometry (F). W, weak fluorescence, represents cells with weaker phagocytic ability; S, strong fluorescence, represents cells with stronger phagocytic ability. POS-LB-positive cells (G) and cells with strong fluorescence (H) were quantified. Scale bar, 10 μm. * *p* < 0.05, ** *p* < 0.01, *** *p* < 0.001, **** *p* < 0.0001.
